# Parkinsonian Symptomatology May Correlate with CT Findings before and after Shunting in Idiopathic Normal Pressure Hydrocephalus

**DOI:** 10.4061/2010/201089

**Published:** 2010-03-10

**Authors:** Mitsuaki Ishii, Toshio Kawamata, Ichiro Akiguchi, Hideo Yagi, Yuko Watanabe, Toshiyuki Watanabe, Hideaki Mashimo

**Affiliations:** ^1^Department of Rehabilitation Science, Kobe University Graduate School of Health Sciences, Kobe 654-0142, Japan; ^2^Department of Physical Therapy, Bukkyo University School of Health Science, Kyoto 603-8301, Japan; ^3^Center of Neurological and Cerebrovascular Disease, Koseikai Takeda Hospital, Kyoto 600-8558, Japan; ^4^Department of Physical Therapy, Maizuru Municipal Hospital, Kyoto 625-0035, Japan

## Abstract

We aimed to investigate the characteristics of Parkinsonian features assessed by the unified Parkinson's disease rating scale (UPDRS) and determine their correlations with the computed tomography (CT) findings in patients with idiopathic normal pressure hydrocephalus (iNPH). The total score and the scores for arising from chair, gait, postural stability, and body hypokinesia in the motor examination section of UPDRS were significantly improved after shunt operations. Stepwise multiple regression analysis revealed that postural stability was the determinant of the gait domain score of the iNPH grading scale. The canonical correlation analysis between the CT findings and the shunt-responsive Parkinsonian features indicated that Evans index rather than midbrain diameters had a large influence on the postural stability. Thus, the pathophysiology of postural instability as a cardinal feature of gait disturbance may be associated with impaired frontal projections close to the frontal horns of the lateral ventricles in the iNPH patients.

## 1. Introduction

Symptoms of idiopathic normal pressure hydrocephalus (iNPH) are potentially reversible if diagnosed and treated in the early stage of the disease [1, 2], and these symptoms may be alleviated by shunt operations. Although iNPH is referred to as “treatable dementia,” gait disturbance occurs earlier and is usually the most frequent symptom; improvement after shunt operation is more likely to occur in this symptom than the other symptoms [3]. Thus, it has been proposed that iNPH be also recognized as a “treatable gait disorder” [4]. Gait disturbance in iNPH is characterized by petit-pas gait, magnetic gait, and broad-based gait [5]. It is believed that iNPH is one of the causes of higher-level gait disorders characterized by a combination of the features of postural instability, hypokinesia, and gait ignition failure [6].

It has also been reported that patients with iNPH exhibit characteristic radiographic abnormalities [7]. Computed tomography (CT) or magnetic resonance imaging (MRI) show enlarged ventricles, frequent white matter lesions [8], increased sylvian fissures space, decreased superior convexity, and medial subarachnoid space; further, in some patients, focally dilated sulci were observed over the convexity of the hemisphere [7]. More recently, decreased diameters of the midbrain have been reported [9]. A few studies report the correlation between gait disturbances and radiographic abnormalities in iNPH [9, 10]. However, whether the severity of the Parkinsonian features can be assessed by the radiographically detected morphologic alterations remains to be determined. 

In addition to characteristic gait disturbances, patients with iNPH exhibit parkinsonism [11–13]. Thus, the unified Parkinson's disease rating scale (UPDRS), which is widely used in the clinical assessment of Parkinson's disease [14], may be used for the evaluation of iNPH. However, investigations on the evaluation of the characteristics of the parkinsonism by using the UPDRS and the correlation between the rating of this scale and the radiographic abnormalities observed in iNPH have not been reported.

To define the characteristic signs and symptoms, an improvement in the symptoms should be observed following a shunt operation. However, previous studies on the correlation between movement disorders and radiographic abnormalities in iNPH have not considered the importance of the above feature.

Our aims were (1) to investigate the characteristics of Parkinsonian features, (2) to explore whether a relationship exists between the parkinsonism, especially those assessed by the UPDRS, and the morphologic changes observed radiographically; (3) and to investigate the correlation between the gait domain score of the iNPH grading scale (iNPHGS) [15] and the motor score of the UPDRS in patients diagnosed with definite iNPH.

## 2. Methods

### 2.1. Subjects

On the basis of clinical guidelines for iNPH, 13 participants (11 males and 2 females) were diagnosed with definite iNPH [4]. They were admitted to the neurological clinic at the Department of Neurology of Koseikai Takeda Hospital or the Department of Neurosurgery of Maizuru Municipal Hospital between August 2004 and January 2006. Their ages ranged from 66 to 81 years with a mean age of 77.0 years (standard deviation (SD) 4.3). All the subjects were informed about the details of the study, and they agreed to participate and provided written informed consent. All the procedures in this study were performed according to the clinical study guidelines of the local ethics committee and were approved by the internal review board.

The clinical guidelines for iNPH propose 3 levels of diagnosis: possible, probable, and definite iNPH. The diagnosis of “possible” iNPH is based on the presence of 1 or more classical symptoms (gait disturbance, cognitive impairment, and urinary incontinence), ventricular dilation (Evans index: maximal width of frontal horns/maximal width of inner skull >  0.31) with closing sulci at high convexity, and clear cerebrospinal fluid (CSF) with a normal CSF pressure in middle-aged and elderly patients. The diagnosis of “probable” iNPH is based on improved gait after the CSF tap test or continuous CSF drainage. The diagnosis of “definite” iNPH is based on the improvement of symptoms after a CSF shunt operation [4]. We excluded patients with NPH secondary to disorders such as subarachnoid hemorrhage.

The Evans index of all patients was more than 0.31. The MRIs of all patients revealed increase in the width of the sylvian fissure and reduction in the width of the cortical sulcal space in the superior convexity [7]. All patients showed frontal release signs such as sucking, grasping, or palmomental reflex. The patients took no medications including levodopa or dopamine agonists before undergoing ventriculoperitoneal shunt (VP shunt) operations. We employed a Codman-Medos adjustable valve for the shunt system. 

## 3. Procedure and Data Analyses

### 3.1. Assessment of Parkinsonism

The Parkinsonian features were assessed by a physical therapist, in terms of the subitems and the total scores of the motor examination section of the UPDRS and the iNPHGS. The best score after 3 measurements was used as the final score. The motor examination section of the UPDRS comprises 14 items. All the items are graded on a 5-point scale, from 0 to 4 [14]. The severity of gait disturbance was semiquantified according to the iNPHGS gait domain score [16]. The iNPHGS gait domain is graded as 0 (normal), 1 (dizziness or awareness of gait disturbance), 2 (gait disturbance not requiring aid), 3 (inability to walk without using a stick, hand-rail, or walker), and 4 (complete inability to walk). It has been reported that (1) the iNPHGS gait domain score significantly correlates with the timed up-and-go test (TUG) and gait status scale scores, and (2) the gait status scale scores significantly improve in patients whose iNPHGS scores improved after CSF tapping but not in those whose iNPHGS scores did not improve after CSF tapping [15]. Here, we investigated the associations between the preoperative iNPHGS gait domain scores and those of the motor examination section of the UPDRS. We performed the postoperative assessments of parkinsonism in the first week after the VP shunts.

### 3.2. Evaluation of Radiographic Abnormalities

**Figure 1:**
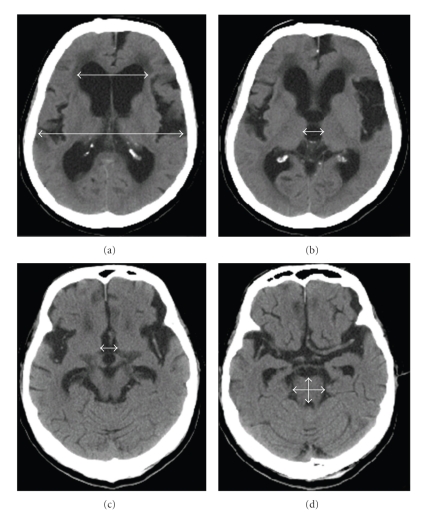
Measurements for iNPH patients on CT scans. (a) Evans index (ratio of maximum width of the frontal horns to the maximum width of the inner table of the cranium); (b) maximum width of the third ventricle at the foramen of Monro (interventricular foramen); (c) maximum width of the third ventricle at the superior colliculus; (d) anteroposterior (vertical) and left-to-right (horizontal) diameters of the midbrain at pontomesencephalic junction.

We performed CT examinations pre- and postoperatively. We investigated the morphologic changes by calculating the values of the Evans index from the CT scans, which are presented in Figure 1(a), the maximum third ventricular width and the midbrain size (anteroposterior and left-to-right diameters). The midbrain diameters were measured at the pontomesencephalic junction (Figure 1(d)). The width of the third ventricle was measured at the levels of the superior colliculus and the foramen of Monro (interventricular foramen) (Figures 1(b) and 1(c)). Measurement of these distances on the CT scans was performed with the Xeron PACS system (Xeron healthcare corp., Korea). Each measurement was repeated 3 times, and the average of these triplicate measurements was recorded.

## 4. Statistical Analysis

Statistical analyses were performed with the software, statistical package for social sciences (SPSS) version 17.0 for Windows. Wilcoxon signed rank tests were used to compare pre- and postoperative values of the total scores of the motor examination section in the UPDRS. The effects of VP shunt on the scores for the subitems of the motor examination in the UPDRS, the gait domain of iNPHGS, and the CT findings were analyzed using 2-way analysis of variance with repeated measures followed by Bonferroni post hoc comparisons. Stepwise multiple regression analysis was conducted to determine the association between the preoperative scores of the gait domain of the iNPHGS and those of motor examination section of the UPDRS. We also examined the correlations between the preoperative parkinsonism and the radiographic abnormalities by using the canonical correlation analysis. CT finding was treated as a multivariate composite variable represented by the combined influences of the five CT measures shown above and was assessed by examining its relation to a parkinsonism construct as measured by UPDRS. The critical value for statistical significance was set at  *P* < .05 for univariate and multivariate analyses.

## 5. Results

Before the VP shunts, the gait domain scores of the iNPHGS were at level 2 in 4 patients, at level 3 in 7 patients, and at level 4 in 2 patients. These scores changed to level 1 in 5 patients and level 2 in 8 patients after the VP shunts. The iNPHGS gait domain scores of all the patients improved after the VP shunts. 

Severe impairment was recorded for the subitems of arising from a chair, gait, and postural stability before the VP shunts. In more than 80% of the patients, the severity of these subitems was graded at higher severity than grade 2. In contrast, the severity of the subitems tremor, rigidity, bradykinesia of extremities, and posture was graded at lower degree of severity than grade 2.

After the operations, the iNPHGS gait domain scores, the subitems (arising from a chair, gait, postural stability, and body hypokinesia), and the total scores of the motor examination section of the UPDRS improved significantly (*P* < .05). There were no significant changes in the other subitems of the motor examination section of the UPDRS (Table 1). 

The results of stepwise multiple regression analysis to assess the association in the scores between the iNPHGS and UPDRS as the dependent variables are shown in Table 2. We used the following independent variables to avoid the problems caused by multicollinearity: tremor at rest, rigidity, finger taps, hand movements, rapid alternating movements of hands, leg agility, arising from a chair, postural stability, and body hypokinesia. Postural stability in the UPDRS was the only factor that revealed to be a determinant of the gait domain score of the iNPHGS (*P* < .05). 

As seen in Table 3, the postoperative values of Evans indices and the maximum widths of the third ventricle measured at the levels of the superior colliculus were markedly decreased (*P* < .05), but those measured at the levels of the foramen of Monro and the midbrain diameters did not significantly change as compared to the preoperative values. 

In canonical correlation analysis, the correlation between the following independent variables was used to reduce the impact of multicollinearity: the subitem scores (arising from a chair, postural stability, and body hypokinesia) and total scores of the motor examination section in the UPDRS. In the first canonical variates of canonical correlation analysis (canonical correlation coefficient = 0.996, *P* < .045), the Evans index had a large influence on the postural stability (Table 4).

## 6. Discussion

### 6.1. Characteristics of Parkinsonism in iNPH

The clinical guidelines for iNPH generally recommend the timed up-and-go (TUG) test for the assessment of disease severity [4]. However, some iNPH patients with severe postural instability are unable to perform the TUG test. In our study, several items of the UPDRS motor examination section proved to be useful in the evaluation of the clinical severity of the characteristic parkinsonism in iNPH, and the patients showed actual improvements in those items after undergoing the VP shunts (Table 1). These shunt-responsive items can reflect the characteristics of the parkinsonism.

The gait domain scores of the iNPHGS were significantly correlated with the scores of postural stability in the motor examination section of the UPDRS (*P* < .001) (Table 2). The high association between the improvement in the postural stability score and in the Evans index after the VP shunt and our results indicate that postural stability, a subitem of the UPDRS motor examination section, might be a specific feature closely related to the gait disturbance in iNPH and that the postural instability may be a cardinal feature of iNPH gait.

Knutsson and Lying-Tunell suggested that the gait disturbances in NPH are similar to gait apraxia [16]. Bugalho and Guimaraes reported that the response of postural instability to CSF tap test was weaker than that of hypokinesia and did not correlate with that of step length or gait velocity in iNPH [17]. However, consistent with the findings from other studies, our results indicate that the gait disturbance in iNPH may be predominantly associated with postural instability rather than hypokinesia [5, 18]. Therefore, we speculate that the short-steppage gait in iNPH may be associated with postural instability.

### 6.2. Improvement in Radiographic Findings

Mocco et al. reported significant increases in the anteroposterior and the left-to-right midbrain diameters but no significant change in the size of the ventricles after shunt operation, stating that gait disturbance in iNPH may be strongly associated with midbrain atrophy [19]. However, in our patients with definite iNPH, the dilatation of the lateral and third ventricles at the levels of the superior colliculus was significantly improved, but the midbrain diameters did not change significantly after a VP shunt.

Our results indicate that CSF loss improved the dilatation of the lateral and third ventricles at the level of the superior colliculus and that these morphologic alterations and not the decreased midbrain diameters are the characteristic features in iNPH.

### 6.3. Correlation between Parkinsonism and Radiographic Abnormalities

Lee et al. reported that the reduction in the maximal anteroposterior diameter of the midbrain was significantly correlated with the severity of gait disturbance, and no significant correlation was observed between gait disturbance and the changes in the diameter of the lateral or third ventricle [9]. However, the results of canonical correlation analysis showed that the postural instability was a cardinal feature of gait disturbance in iNPH, and it was influenced by ventricle widths rather than midbrain diameters. They also suggested that the gait disturbances may be associated with postural instability in iNPH as well as in progressive supranuclear palsy (PSP) or advanced Parkinson's disease (PD) [20] and that the neuronal dysfunction in pedunculopontine nucleus (PPN), the major component of the mesencephalic locomotor region, may be important in the pathophysiology of locomotor and postural disturbances. Although loss of neurons in the PPN was reported in PSP and advanced PD [20], there are no reports of the PPN pathology in iNPH to our knowledge. Our results indicate that the lesions involved in the gait disturbance in the case of iNPH patients are not specific to the midbrain (Table 4).

Our results indicate that Evans index had a large influence on the postural stability (Table 4). In addition, patients with iNPH are known to have frequent frontal release signs, and an association has been reported between frontal cognitive impairment and gait disturbance [21]. In this context, an association may exist between frontal lobe dysfunction and the postural instability in iNPH. It is possible that the postural instability in iNPH is associated with impaired frontal projections descending close to the frontal horns of the lateral ventricles toward the reticular formation in the tegmentum of the brainstem [22].

Cerebral blood flow (CBF) studies in iNPH have shown a decrease in the BG blood flow, and this decrease has been found to be correlated with gait disturbance [23, 24]. Pathological changes in the BG have been reported in postmortem brains of the iNPH patients [25]. However, it remains controversial whether the mechanism of the gait disturbance can be explained only by the functional defect in the BG loop. Stolze et al. stated that the poor response to external cues for improving gait velocity and step length indicate that the BG-supplementary motor area (SMA) loop may not be a major lesion causing the gait disturbance in iNPH [5].

 Our study has several limitations. First, we used a small sample size. Second, the retrospective design of our study may introduce a selection bias. Third, since the assessments of parkinsonism were semiquantitative, the accuracy of assessments may be poor. Further study using kinetic analysis with more iNPH patients is needed to investigate the gait disturbance or postural instability. Finally, although CT remains the standard examination for postoperative imaging in a clinical setting to investigate the brain morphological alterations in iNPH, the image resolution obtained in CT is markedly lower than that in MRI.

## Figures and Tables

**Table 1:** Comparison between the grades of the Parkinsonian features before and after ventriculoperitoneal shunt operation.

	Pre VP shunt	Post VP shunt	*P* value
	(Mean ± SD)	(Mean ± SD)	
iNPH grading scale gait domain	2.85 ± 6.9	1.62 ± 0.86	.001

UPDRS motor examination subitems			
Speech	0.92 ± 0.80	0.54 ± 0.66	.200
Facial expression	1.00 ± 0.91	0.69 ± 0.75	.305
Tremor at rest	0.46 ± 0.78	0.15 ± 0.38	.305
Action tremor of hands	0.46 ± 0.66	0.23 ± 0.44	.442
Rigidity	0.85 ± 0.80	0.38 ± 0.65	.124
Finger taps	1.15 ± 0.90	0.77 ± 0.73	.200
Hand movements	1.00 ± 0.82	0.69 ± 0.75	.305
Rapid alternating movements of hands	1.15 ± 0.80	0.92 ± 0.86	.442
Leg agility	0.85 ± 0.69	0.69 ± 0.75	.608
Arising from chair	2.69 ± 1.25	1.23 ± 0.93	.001
Posture	1.31 ± 0.48	1.00 ± 0.00	.305
Gait	2.54 ± 0.88	1.00 ± 0.56	.001
Postural stability	2.54 ± 1.05	1.31 ± 0.75	.001
Body bradykinesia and hypokinesia	2.15 ± 0.80	1.46 ± 0.52	.021

UPDRS motor examination total score	19.08 ± 5.66	12.00 ± 7.07	.008*

VP shunt: ventriculoperitoneal shunt; iNPH: idiopathic normal pressure hydrocephalus; UPDRS: unified Parkinson's disease rating scale; *P* value: *P* value by 2-way analysis of variance with repeated measure, *P value by Wilcoxon signed rank test. The total number of patients was 13.

**Table 2:** Item in the motor examination of unified Parkinson's disease rating scale associated with the gait domain of idiopathic normal pressure hydrocephalus grading scale.

	Standardized partial regression coefficient	Partial regression coefficient	95%CI	*P* value
Postural stability	0.815	0.535	0.3–0.81	.001

*P* value by stepwise multiple regression analysis. The total number of patients was 12.

**Table 3:** Effect of ventriculoperitoneal shunt operation on the computed tomography findings.

	Pre VP shunt	Post VP shunt	*P* value
	(Mean ± SD)	(Mean ± SD)	
Evans index	0.38 ± 0.047	0.33 ± 0.43	.001
Third ventricle width at superior colliculus	10.55 ± 1.80	8.14 ± 2.03	.037
Third ventricle width at foramen of Monro	12.87 ± 2.00	11.24 ± 2.43	.155
Anteroposterior diameter of midbrain	20.86 ± 2.03	22.47 ± 1.77	.159
Left-to-right diameter of midbrain	29.64 ± 2.82	29.59 ± 2.14	.967

*P *value by 2-way analysis of variance with repeated measure. The total number of patients was 12.

**Table 4:** Canonical correlations between the grades of the Parkinsonian features responding to the ventriculoperitoneal shunt operation and computed tomography findings.

	First canonical variate	
	Correlation	Coefficient
CT finding set		
Third ventricle width at superior colliculus	−0.23	0.908
Third ventricle width at foramen of Monro	−0.612	−1.011
Anteroposterior diameter of midbrain	0.094	0.529
Left-to-right diameter of midbrain	−0.162	−0.504
Evans index	−0.814	−0.564
Percent of variance	0.225	
Redundancy index	0.223	
Parkinsonism set (assessed by UPDRS)		
Total score of motor examination	−0.59	−1.151
Subitem score of postural stability	−0.592	0.624
arising from chair	−0.499	−0.605
body bradykinesia and hypokinesia	0.356	1.09
Percent of variance	0.27	
Redundancy index	0.266	
Canonical Correlation	0.996	

CT: computed tomography; *n*: number of subjects; UPDRS: unified Parkinson's disease rating scale; Percent of variance = average squared loading (*L* ^2^); Redundancy index = average *L* ^2^.  canonical *R* ^2^. The total number of patients was 11.
